# Deep learning architectures for diagnosing the severity of apple frog-eye leaf spot disease in complex backgrounds

**DOI:** 10.3389/fpls.2023.1289497

**Published:** 2024-01-08

**Authors:** Bo Liu, Hongyu Fan, Yuting Zhang, Jinjin Cai, Hong Cheng

**Affiliations:** ^1^ College of Information Science and Technology, Hebei Agricultural University, Baoding, China; ^2^ Hebei Key Laboratory of Agricultural Big Data, Baoding, China; ^3^ College of Mechanical and Electrical Engineering, Hebei Agricultural University, Baoding, China

**Keywords:** apple disease, severity estimation, deep learning, frog eye leaf spot, two-stage method

## Abstract

**Introduction:**

In precision agriculture, accurately diagnosing apple frog-eye leaf spot disease is critical for effective disease management. Traditional methods, predominantly relying on labor-intensive and subjective visual evaluations, are often inefficient and unreliable.

**Methods:**

To tackle these challenges in complex orchard environments, we develop a specialized deep learning architecture. This architecture consists of a two-stage multi-network model. The first stage features an enhanced Pyramid Scene Parsing Network (L-DPNet) with deformable convolutions for improved apple leaf segmentation. The second stage utilizes an improved U-Net (D-UNet), optimized with bilinear upsampling and batch normalization, for precise disease spot segmentation.

**Results:**

Our model sets new benchmarks in performance, achieving a mean Intersection over Union (mIoU) of 91.27% for segmentation of both apple leaves and disease spots, and a mean Pixel Accuracy (mPA) of 94.32%. It also excels in classifying disease severity across five levels, achieving an overall precision of 94.81%.

**Discussion:**

This approach represents a significant advancement in automated disease quantification, enhancing disease management in precision agriculture through data-driven decision-making.

## 1 Introduction

Leaves are critical factors in the process of sunlight interception and its subsequent conversion into biochemical energy, essential for plant growth and health (Sala et al., 2015). Diseases affecting apple tree leaves, such as the apple frog-eye leaf spot caused by fungi of the genus Cercospora, can have a detrimental impact on both the yield and quality of apple crops (Venkatasubbaiah et al., 1991; Abbasi et al., 2019). Accurate assessment of disease severity is therefore imperative for effective disease management (Liu et al., 2022). Bock et al. (2010) have shown automated disease diagnosis through computer vision technologies can maintain consistency with traditional human observations while offering significant advantages in efficiency.Additionally, automated disease diagnosis can be optimized over time with more training data. While, manual visual assessment or measurement of the percentage of leaf area affected in orchards still relies heavily on human labor, characterized by low efficiency (Bock et al., 2010). Automated disease diagnosis through computer vision technologies not only maintains consistency with traditional human observations but also offers significant advantages in efficiency Bock et al. (2022). Moreover, it is important to emphasize the role of accurate disease severity estimation in determining the right amount of pesticide. Automated systems contribute to this accuracy, especially in early disease stages, and thus can significantly reduce pesticide usage Patil et al. (2011).

In recent years, significant strides have been made in the development of deep learning-based algorithms for automatic segmentation and recognition of leaf diseases. Initial efforts, such as the semantic segmentation model by Lin et al. (2019) and the traditional threshold segmentation methods by Esgario et al. (2020), focused on controlled environments with simple backgrounds. These models have shown high accuracy rates, such as 96.08% in the case of Lin et al. (2019) and 84.13% for Esgario et al. (2020). However, their performance is often compromised when applied to real-world agricultural settings due to the complexity of natural backgrounds and the diversity of disease symptoms (Thakur et al., 2022; Wang et al., 2022b). In response to these challenges, recent research has pivoted toward models that can adapt to the complexities of field images. One promising approach is the use of multi-stage models, which significantly enhance disease recognition by first segmenting leaves and then refining the identification of disease spots within those segmented areas Wang et al. (2021). Despite these advancements, certain issues persist, particularly in handling intricate image contexts. For instance, while Liu et al. (2022) and Zhu et al. (2023) excel in leaf segmentation, they struggle with detecting smaller lesions. Similarly, Tassis et al. (2021) introduced instance segmentation to improve background handling but at the expense of increased model complexity.

To address these limitations, this paper introduces a novel two-stage approach for estimating disease severity in complex environments. In the first stage of our approach, we introduce L-DPNet, a leaf segmentation model that incorporates deformable convolutions into the PSPNet architecture. These deformable kernels adapt dynamically to various leaf shapes and occlusions, enlarging the receptive field to capture more contextual information. Through end-to-end learning, the model adjusts to leaf shape variations without manual intervention. As a result, L-DPNet not only addresses the shortcomings of existing methods but also enhances segmentation accuracy, setting a precise foundation for disease diagnosis. In the second stage of our approach, we employ D-UNet, an enhanced U-Net architecture tailored for disease segmentation. Building on the strengths of traditional U-Net models, D-UNet incorporates several key improvements. A batch normalization layer is integrated to mitigate overfitting, particularly on complex lesion patterns, ensuring robust generalization. To refine segmentation quality, especially for small, dense spots, bilinear upsampling replaces transposed convolution, eliminating checkerboard artifacts. Additionally, the model addresses the class imbalance between diseased and healthy pixels by incorporating Focal loss into the objective function. This focuses the training on hard-to-classify examples, thereby boosting the model’s performance on the under-represented diseased class.

The main contributions of this paper are as follows:

1. We introduce a two-stage approach for comprehensive disease analysis in apple leaves, starting with L-DPNet for leaf segmentation. L-DPNet is a specialized model that enhances the existing PSPNet by incorporating deformable convolutions and optimizing pyramid pooling layers for computational efficiency. This first stage sets the foundation for the subsequent disease spot segmentation.
2. Alongside L-DPNet, We present D-UNet, an optimized U-Net architecture for disease spot segmentation. It builds on the VGG16 architecture and includes batch normalization and bilinear interpolation to improve segmentation quality and mitigate overfitting.
3. We integrate L-DPNet and D-UNet into a unified framework, achieving 98.90% accuracy in leaf segmentation, 99.89% in lesion segmentation, and 94.81% in disease severity classification. This provides a robust tool for apple leaf disease diagnosis and treatment.

The rest of this paper is arranged as follows: Section 2 introduces Materials and Methods, including Data collection/Datasets pre-processing and Deep-Learning Algorithms. Section 3 introduces Experiment and result analysis, many experiments were carried out.Finally, some conclusions are drawn in Section 4.

## 2 Materials and methods

### 2.1 Data collection and pre-processing

**Figure 1:**
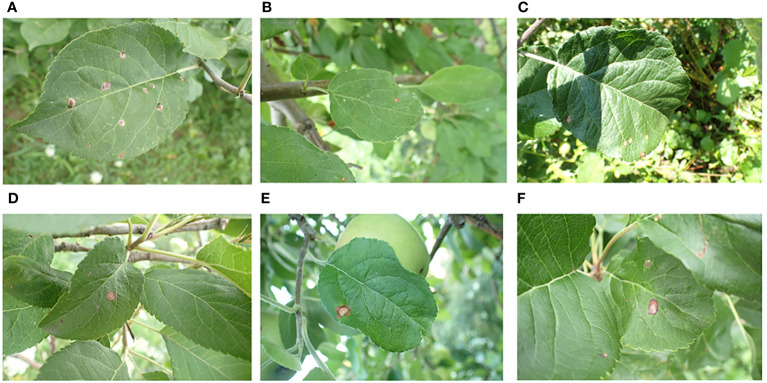
Representative image of apple leaves in a complex environment: **(A)** Green grass on the ground; **(B)** Tree trunk; **(C)** Shadow; **(D)** Healthy leaves; **(E)** Apple fruit; **(F)** Obstruction.

The apple leaf image dataset used in this study is sourced from the public dataset Plant Pathology 2021, which supplements the Plant Pathology 2020 dataset (Thapa et al., 2020), originally provided by the 2020 Kaggle Plant Pathology competition.It contains RGB images of 4 types of diseases and healthy leaves of apple, with a total of 23,000 images. The dataset can be downloaded from the Kaggle website ^1^ ^,2^.The images in the dataset were captured using professional cameras with varying focal lengths, at a resolution of 4,000 ×2,672 pixels. Each image focuses on a single leaf but also contains complicating background elements, such as grass, healthy leaves, trunks, fruit, and variations in light exposure. These complex background elements helped train the model to handle real-world environmental variables, thereby improving its performance. **Figure 1** displays several examples of such images. We specifically focused on the apple frog-eye leaf spot disease, selecting 1,372 images with complex backgrounds. These images are randomly divided into training, validation and test sets at a ratio of 8:1:1, with 1,096, 138 and 138 images in each set.

**Figure 2:**
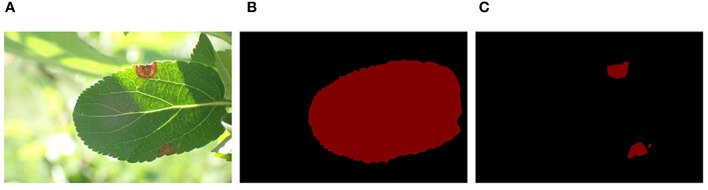
Leaf and disease labels in the dataset: **(A)** Original images; **(B)** Leaf labels; **(C)** Disease spot labels.

For deep learning, it is crucial that the image dataset be manually annotated before training the model (Zou et al., 2021).Since the original dataset only provides image-level category labels, while our research goal is to estimate the severity of the disease on the leaves based on the results of image semantic segmentation, specifically pixel-level classification of images, the labels provided in the original dataset are not suitable for our task. Therefore, we manually label using the pixel-level segmentation tool LabelMe (Kurtser et al., 2016).The image annotation results match the annotation content, as shown in **Supplementary Figure 1**.The annotation files are in JSON format and can be converted into PNG (portable network graphics) image files for training purposes. The labeling results for the leaves and lesions are illustrated in **Figure 2**.

**Figure 3:**
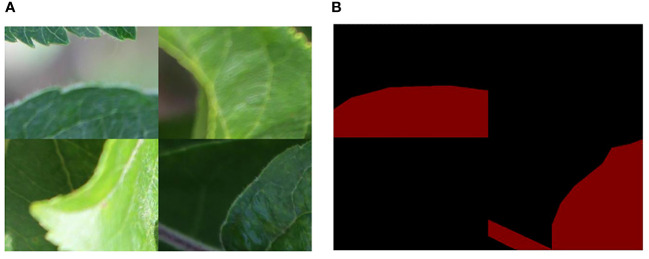
Mosaic method for data augmentation: **(A)** Enhanced rgb image; **(B)** Enhanced mask image.

To enrich the background information of the detected object, the Mosaic image augmentation method was applied (Bochkovskiy et al., 2020; Chen et al., 2022; Xing and Chen, 2022). This label-preserving method, with strong generalization capabilities, randomly selects four images and performs random cropping and collaging, thereby enriching the background of the detected objects. The annotation files for the corresponding collaged images can be obtained by modifying their ground truth metadata. These spliced images were then fed into the model for learning. The effect of mosaic data augmentation is shown in **Figure 3**. After data augmentation, a total of 2,192 target images are obtained for model training. The specific dataset information is shown in **Table 1**.

**Table 1:** Dataset information.

Level	Training		Validation Test	Test	Total	
	Original	Augmented			Original	Augmented
Level 1	238	476	36	38	312	550
Level 2	241	482	35	30	306	547
Level 3	219	438	33	30	282	501
Level 4	196	392	25	30	251	447
Level 5	202	404	9	10	221	423
Total	1096	2192	138	138	1372	2468

### 2.2 Diagnosing the severity of the disease

Disease severity classification serves as one of the bases for developing prevention, control, and treatment strategies. Currently, no unified classification standard exists for the severity of apple frog-eye leaf spot disease. According to the literature, one common method is to calculate the ratio of the diseased area to the total leaf area on the same leaf. This method forms the basis for accurately estimating the severity of crop diseases in a given region (Bock et al., 2010). Therefore, this study adopts this method, using the ratio of the diseased area to the total leaf area on the same leaf as the basis for disease severity classification. The calculation Formula 1 is presented as follows:


(1) $$L=\frac{S_{Leaf}}{S_{Disease}}$$

where *S_Leaf_* represents the segmented leaf area calculated as the sum of leaf pixel counts, *S_Disease_* represents the segmented disease spot area calculated as the sum of disease spot pixel counts, and *L* represents the ratio of the disease spot area (*S_Disease_*) to the total leaf area (*S_Leaf_*).

Based on the reference of relevant disease grading standards and suggestions from plant protection experts (Liu et al., 2022), the severity of apple frog eye leaf spot disease is divided into five levels from level 1 to level 5 as shown in **Table 2**. The severity of the disease is determined based on the degree of damage to apple leaves, using the proportion of apple frog eye leaf spot disease damage to the total leaf area. Level 1 refers to damage below 0.95%; Level 2 refers to 0.95%-1.50% damage; Level 3 refers to 1.50%-2.15% damage; Level 4 refers to 2.15%-3.00% damage; Level 5 refers to damage equal to or greater than 3.00%. The complete severity estimation process and workflow are shown in **Supplementary Figure 2**.

**Table 2:** Criteria for disease severity classification based on the ratio of diseased area to leaf area.

Disease severity level	Ratio *L* of disease area to total leaf area
Level 1	0%<= *L<* 0.95%
Level 2	0.95%<= *L<* 1.50%
Level 3	1.50%<= *L<* 2.15%
Level 4	2.15%<= *L<* 3.00%
Level 5	*L >*= 3.00%

### 2.3 Methods

**Figure 4:**
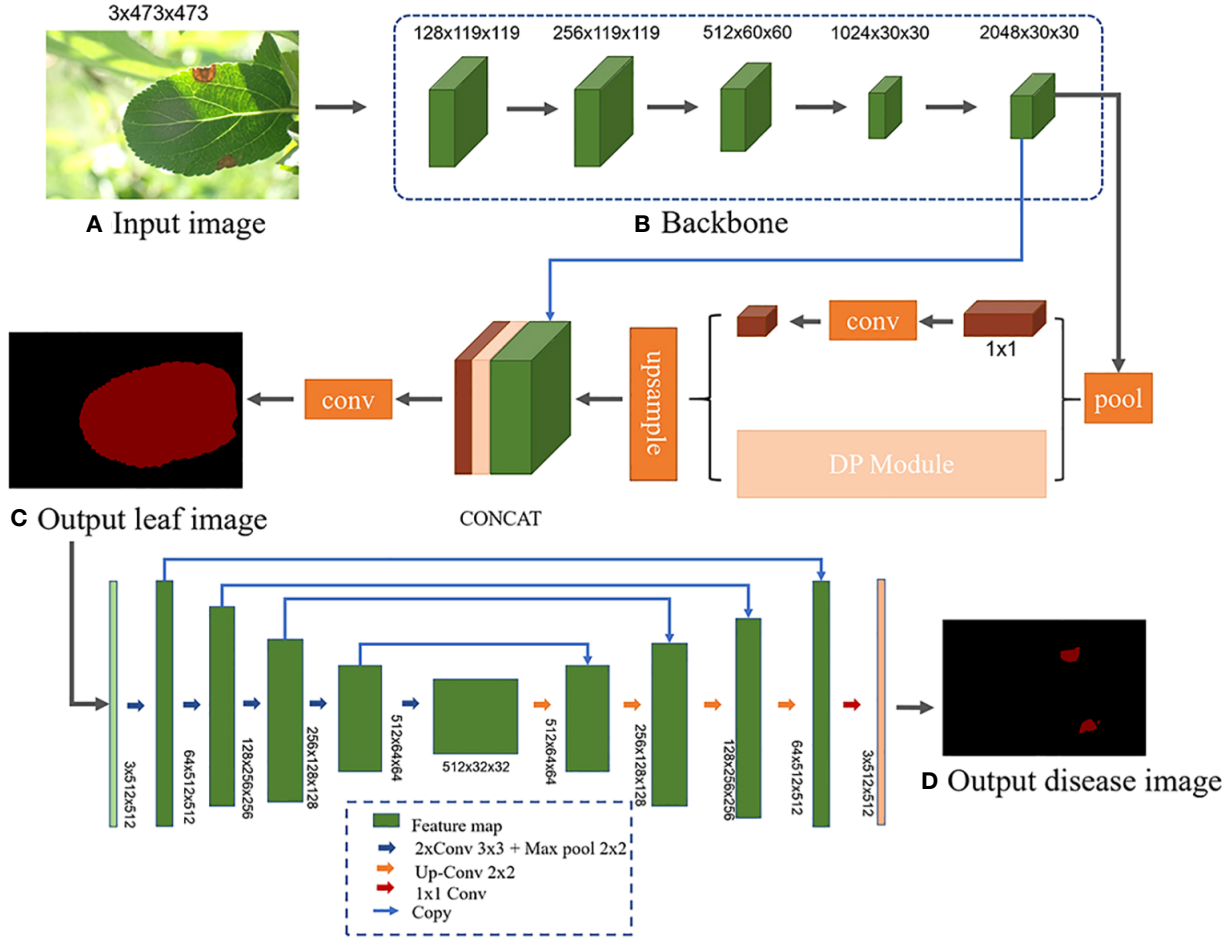
L-DPNet+D-UNet network model architecture. **(A)** Input image. **(B)** Backbone. **(C)** Output leaf image. **(D)** Output disease image.

Different semantic segmentation models possess distinct network architectures, which can influence the segmentation accuracy of leaves and disease spots. Utilizing the same segmentation model for both stages might compromise the model’s feature extraction capability due to the differing segmentation objectives (Wang et al., 2022a). Therefore, a more suitable semantic segmentation model is chosen for each stage, tailored to the specific features to be extracted. Liu et al. (2022) segmented apple tree leaves in complex backgrounds using various deep learning algorithms. Their experimental results showed that the PSPNet model excelled in leaf segmentation, while the UNet model was superior for predicting disease areas. However, there were still some errors in handling occlusions and small spots, leading to incomplete and inaccurate segmentation. Further improvements in accuracy are needed. Moreover, current research on identification and diagnosis of apple frog-eye leaf spot disease remains insufficient, without application to semantic segmentation and severity assessment. Building on their work, this study aims to improve the PSPNet model by incorporating deformable convolutions to segment apple leaves under challenging field conditions. This addresses issues such as low segmentation accuracy arising from factors like occlusion, capture level, and lighting conditions. The segmented results are subsequently fed into D-UNet network for disease spot detection. The severity of apple frog-eye leaf spot disease is then assessed based on the ratio of the segmented leaf area to the disease spot area. The network architecture is depicted in **Figure 4**.

#### 2.3.1 Leaf segmentation based on L-DPNet

The dataset for apple frog-eye leaf spot disease presents several challenges, including varied image acquisition environments, diverse leaf colors and shapes, non-uniform backgrounds, and inconsistent lighting conditions. The PSPNet network, which uses a pyramid pooling module to capture local features at multiple scales, offers a foundation for tackling these issues (Zhao et al., 2017). PSPNet, short for Pyramid Scene Parsing Network, is a convolutional neural network architecture designed for semantic segmentation. The goal of PSPNet is to address scene parsing challenges in semantic segmentation tasks. In complex scene images, the same object may appear at different scales and spatial locations. To correctly segment these objects, the model needs to understand the global contextual information in the image. The pyramid pooling module in PSPNet can capture global contextual information at different scales of the image, enabling both local and global receptive fields to extract multi-scale features for more accurate scene parsing and semantic segmentation. Building on this foundation, this study introduces key improvements tailored to the specific characteristics of apple leaves in various environmental settings. These improvements enhance both shallow and deep feature extraction capabilities of the core pyramid pooling module. As a result, we develop an improved model, referred to as L-DPNet, which is subsequently employed for accurate leaf segmentation.

**Figure 5:**
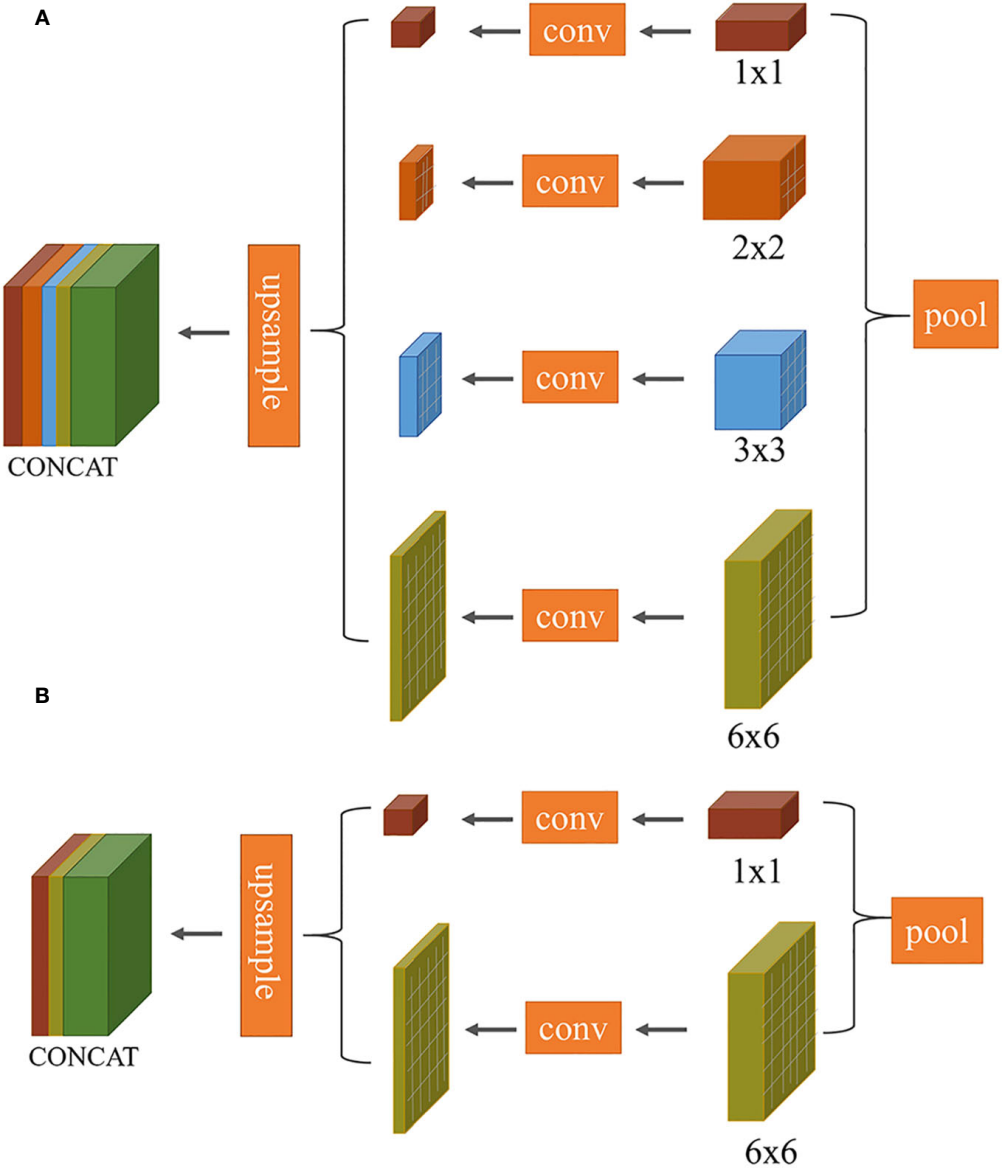
Comparison of multi-scale convolutional layers between PSPNet and our proposed method: **(A)** PSPNet (pyramid pooling structure); **(B)** Our optimized structure.

**Improvement 1:** The task of segmenting target leaves from the background in this study is essentially a binary classification problem. In the original PSPNet network, the multi-scale pyramid pooling layer introduces computational redundancies, as demonstrated in **Figure 5A**. To streamline this, we made an improvement to the model’s architecture (as illustrated in **Figure 5B**). Specifically, the number of pyramid pooling layers was reduced from four to two, with retained pooling kernel sizes of 1×1 and 6×6.

The 1 × 1 pooling represents the coarsest level of global pooling, integrating global spatial information to generate a single feature map. The 6 × 6 pooling divides the feature map into 6 × 6 sub-regions, where each sub-region undergoes pooling. This allows the model to capture both local and global information. To maintain channel consistency during the subsequent upsampling process, the input feature map first undergoes compression through two different scale pooling layers. This is followed by a 1 × 1 convolution to halve the number of channels. The feature map is then upsampled back to its original dimensions using bilinear interpolation, ensuring that it matches the size of the initial input feature map. The final output feature map is obtained by concatenating these two processed feature maps.

**Improvement 2:** In segmenting leaves affected by apple frog-eye leaf spot disease against a complex background, traditional convolution units sample the input feature map at fixed locations, maintaining a uniform receptive field size across the same convolution layer. Given the complex backgrounds and potential occlusions of target leaves, an adaptive method is required to prevent issues like incomplete leaf segmentation and low accuracy. Typically, the implementation of 2D convolution comprises two steps: 1) sampling the input feature map *x*using a regular grid *R*; 2) multiplying the sampled values by the corresponding weights *w* and then summing. For each position *p*_0_ on the output feature map *y*, we have the calculation Formula 2:


(2) $$y\left(p_{0}\right)=\sum_{p_{n}\in R}w\left(p_{n}\right)x\left(p_{0}+p_{n}\right)$$

where *x*(*p*_0_ + *p_n_*) enumerates different positions on the input feature map, *w* (*p_n_*) denotes the weight values of the convolution kernel, and *y* (*p*_0_) enumerates different positions on the output feature map. Deformable convolution networks address this by allowing each convolution operator to have a learnable offset, adaptively learned from the data (Dai et al., 2017). An offset {Δ*p_n_* | *n* = 1*,…,N*} is added to the regular grid *R*, where *N* = |*R*|. Here, Δ*p _n_* represents the learnable offset at each standard convolution sampling position. Given a position *p _n_* in *R*, the position on the grid becomes *p*_0_ + *p_n_* + Δ*p_n_*, and each output image position is represented as *p*_0_. The convolution expression is Formula 3:


(3) $$y\left(P_{0}\right)=\sum_{p_{n}\in ℛ}w\;\left(p_{n}\right)\cdot x\;\left(p_{0}+p_{n}+\text{Δ}p_{n}\right)$$

After learning, the obtained offsets Δ*p_n_* are typically decimals. The pixel values at the sampling positions *x *(*p*_0_ + *p_n_* + Δ*p_n_*) are then bilinearly interpolated. For notational convenience, let *p* = *p*_0_ + *p_n_* + Δ*p_n_*, which corresponds to the nearest pixel point. The equations for interpolation are Formula 4:


(4) $$x\left(p\right)=\sum_{q_{i}}w_{i}\left(q_{i},p\right)\cdot x\;\left(q_{i}\right),\;w_{\text{i}}\;\left(q_{i},p\right)=ɡ\;\left(q_{i_{x}},p_{x}\right)\cdot g\;\left(q_{i_{y}},p_{y}\right)$$

where *q_i_* enumerates all integer spatial positions in the feature map *x*, specifically the four surrounding integer points of *p*. The bilinear interpolation kernel function *w_i_* (*q_i_,p*) is obtained by multiplying the kernel functions in the *XY* directions. It can be defined using the function *ɡ* (*a,b*) = max(0,1 − |*a* − *b*|).

**Figure 6:**
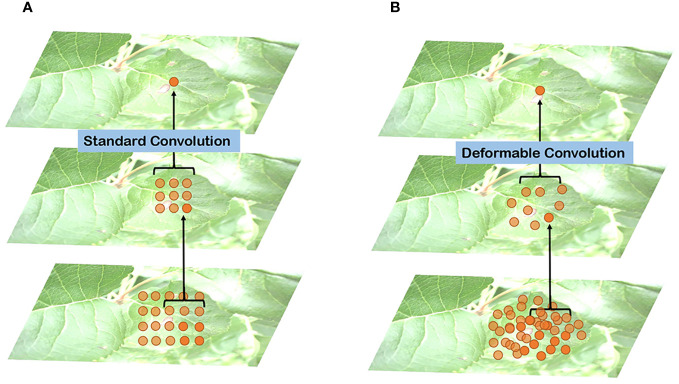
Comparison of receptive field distribution between traditional and deformable convolutions on leaves: **(A)** Traditional convolutions; **(B)** Deformable convolution.

In **Figure 6**, a comparison between standard and deformable convolution for leaf sampling is presented. The receptive field of standard convolution maintains a fixed rectangular shape, in contrast to the polygonal shape exhibited by the deformable convolution’s receptive field. This adaptability in the shape of the receptive field allows the network to better capture the irregular features of leaves. The introduction of deformable convolution enhances the L-DPNet model’s ability to adapt to the unique features of apple leaves in complex natural environments. Given that the shape and structure of apple leaves are often irregular, traditional fixed-shape receptive fields might not adequately capture these details. Moreover, deformable convolution enables the network to adjust the shape of the receptive field adaptively at each position, thus improving the capture of the leaves’ irregular features.

**Figure 7:**
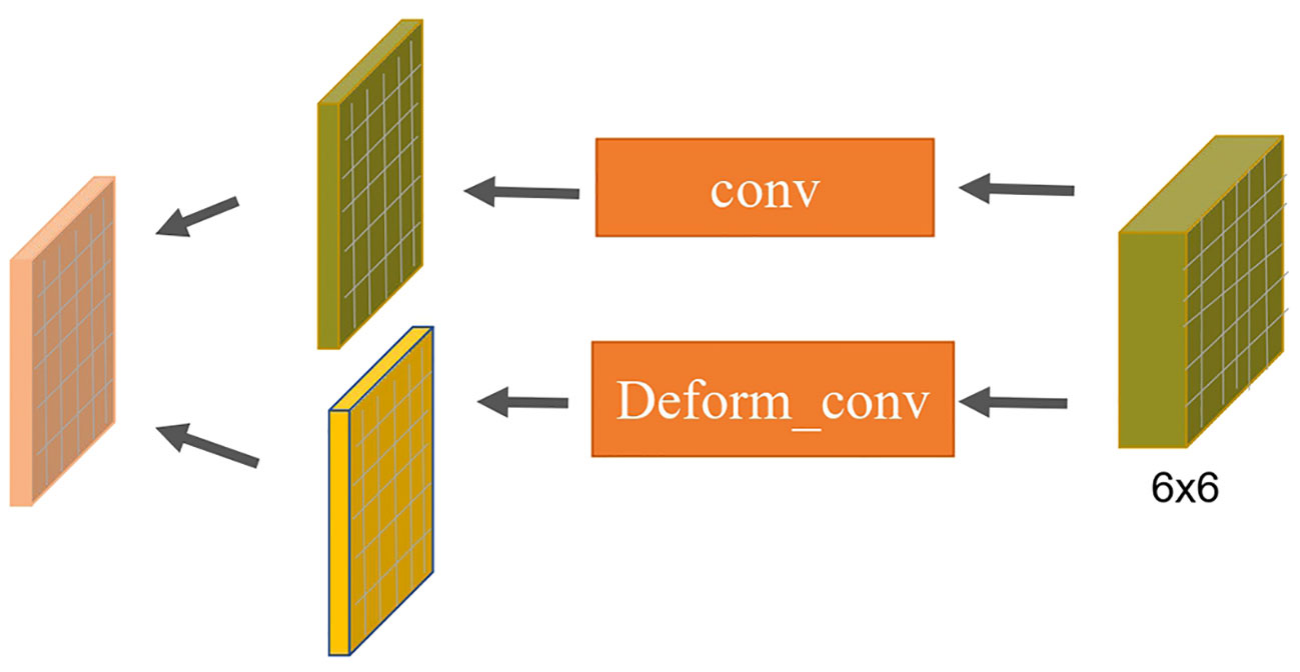
Configuration of deformable convolutions in our L-DPNet model(DP Module).

The decoding phase of the L-DPNet comprises two layers - a 1x1 pooling layer and a DPNet layer as shown in **Figure 7**. After obtaining the 6x6 pooled representation, we incorporate a deformable convolution layer, then element-wise add the resulting features to the convolved feature map to obtain the corresponding feature map. The deformable convolution layer aims to learn more complex features. Essentially, it is a PSPNet model enhanced with deformable convolution.

#### 2.3.2 Segmentation of lesions based on improved U-Net

Due to the inherent structural constraints and limited semantic richness of apple frog eye leaf spot disease, both high-level semantic information and low-level features are crucial for accurate segmentation (Liu et al., 2020). U-Net is a fully convolutional network architecture for medical image segmentation consisting of an encoder and decoder in a u-shaped structure (Long et al., 2015). By fusing shallow and deep feature maps, it combines low-level features such as points, lines, and edge contours with high-level semantics. The shallow feature maps tend to represent basic building units and contain more spatial information, while the deep feature maps encode more semantic information with less spatial resolution. This architecture is especially suitable for segmenting small targets and effectively concatenates high-level semantics with low-level features (Anwar et al., 2018; Liu et al., 2020).

Furthermore, when dealing with limited data, U-Net can achieve satisfactory performance when trained end-to-end after data augmentation (Ronneberger et al., 2015). Therefore, the convolutional neural network employed in the second stage of this study for lesion segmentation is primarily based on U-Net.

To leverage pre-trained models and accelerate training, this study integrates the U-Net architecture with the VGG16 network model (Simonyan and Zisserman, 2014). The VGG16 network itself is a classification network with 16 layers, including 13 convolutional layers, 5 max pooling layers, and 3 fully connected layers. Specifically, the detailed structure, image size, and convolution kernel size are shown in **Supplementary Table 1**. In the Encoder section, the D-UNet model uses the 13 convolutional layers and 4 max pooling layers of VGG16, discarding the 5th max pooling layer and 3 fully connected layers to complete the downsampling feature extraction of the DUNet network. To prevent overfitting, we introduce a batch normalization layer BN (Batch Normalization) before each activation layer ReLU. Through the Encoder part, five preliminary valid feature layers can be obtained, as shown in **Figure 4**. The Decoder part of D-UNet utilizes the five preliminary valid feature layers obtained from the backbone to perform bilinear interpolation upsampling instead of the transpose convolution upsampling used in the original network, and then feature fusion to obtain a final valid feature layer that integrates all features.

The segmentation of lesion regions is essentially a binary classification problem for each pixel. However, the number of pixels in the lesion region is smaller than that in the non-diseased region. This imbalance can lead to lower accuracy for the class with fewer samples, reducing the overall recognition accuracy for the disease region. To mitigate this issue, the D-UNet model employed in this study uses a Focal loss function *FL*(*p_t_*) (Lin et al., 2017), which is defined as Formula 5:


(5) $$FL\;\left(p_{t}\right)=-\;{\left(1-p_{t}\right)}^{\gamma }\log\;\left(p_{t}\right)$$

where *p_t_* represents the probability (confidence) of the predicted class by the model. *γ* is used to adjust the problem of imbalanced samples between difficult and easy classes, and in this study,*γ* is set to 2 to lower the loss of easy samples by a power function. Multiplying with ${\left(1-p_{t}\right)}^{\gamma }$ makes the model more focused on difficult samples.

## 3 Experiment and result analysis

### 3.1 Model training

The hardware platform for the experiments comprises an Intel Core i9-9900X CPU and an NVIDIA GeForce RTX 2080 Ti GPU. The software environment includes a 64-bit Ubuntu 20 system with the PyTorch deep learning framework. **Table 3** lists specific modeling parameters such as batch size for training and validation, base learning rate, and the maximum number of iterations, which are set based on the GPU’s capacity and the dimensions of the sample images.

**Table 3:** Modeling parameters for L-DPNet and D-UNet.

Modeling Parameters	L-DPNet Model	D-UNet Model
Input size	473x473	512x512
Training number of epochs	200	200
Base learning rate	0.0001	0.0001
Image input batch size	16	4
Gamma	0.1	0.96
Number of classes	2	2

The original images have dimensions of 4,000×2,672, necessitating scaling or cropping to fit the model’s input size. This step reduces computational complexity and ensures compatibility with the model’s input layer. While downscaling image size does result in some loss of detail, preprocessing and model training strategies are employed to maintain the accuracy of results, even with smaller input images.

### 3.2 Evaluation metrics

To test the performance of the model used in this study, Precision (%), Recall (%), Mean Intersection over Union (mIoU, %), and average Pixel Accuracy (mPA, %) were selected as the indicators Wang et al. (2020c).

#### 3.2.1 Precision and recall

Our model has two segmentation stages. In both stages, true positives (TP) are pixels correctly identified as the target, false positives (FP) are incorrectly identified pixels, and false negatives (FN) are missed target pixels. In stage one, TP are leaf pixels, FP are background pixels incorrectly marked as leaf, and FN are leaf pixels missed. In stage two, TP are diseased spots correctly identified, FP are healthy leaves incorrectly marked diseased, and FN are missed diseased spots. We evaluate our model’s performance using Precision and Recall as Formulas 6 and 7:


(6) $$\text{Precision}=\frac{TP}{TP+FP}$$

(7) $$\text{Recall}=\frac{TP}{TP+FN}$$

Precision assesses the accuracy in classifying pixels, indicating the likelihood that pixels identified as leaf tissue (first stage) or diseased spots (second stage) are accurately classified, which in turn reduces false positives. Recall measures the model’s capability to detect all relevant pixels, reflecting the probability of correctly identifying all leaf pixels (first stage) and diseased spots (second stage), which helps in minimizing false negatives.

#### 3.2.2 mIoU and mPA

mIoU is a standard metric used to evaluate the performance of image segmentation. It represents the ratio of the intersection area between the input label mask and the prediction result mask to their union area. A larger value of mIoU indicates better segmentation. mPA measures the average Pixel Accuracy across all categories, where a larger value signifies better classification performance by the model. For ease of explanation, let’s assume that the dataset contains *k* + 1 categories. Here, *p_ij_* denotes the number of pixels where category *i* is predicted as category *j*. *p_ii_* represents the number of pixels correctly predicted, while *p_ij_* and *p_ji_* stand for the numbers of false-negative and false-positive pixels, respectively mIoU and mPA as Formulas 8 and 9:


(8) $$\text{mIoU}=\frac{1}{k+1}\sum_{i=0}^{k}\frac{p_{ii}}{\sum_{j=0}^{k}\;p_{ij}+\sum_{j=0\;}^{k}p_{ji}-p_{ii}}$$

(9) $$\text{mPA}=\frac{1}{k+1}\sum_{i=0}^{k}\frac{p_{ii}}{\sum_{j=0}^{k}\;p_{ij}}$$

In the first stage of our model, mIoU measures accuracy in distinguishing leaf versus non-leaf areas; higher values indicate more precise leaf segmentation. mPA assesses success in classifying pixels as leaf or background, with higher values signifying greater accuracy. In the second stage, mIoU is key for assessing precision in differentiating diseased spots versus healthy tissue; higher values reflect more accurate identification of diseased regions. mPA evaluates the effectiveness in classifying pixels as diseased or healthy, where higher values show improved detection of disease spots.

### 3.3 Experiment and analysis

#### 3.3.1 Experimental analysis for leaf segmentation

To evaluate the impact of the number of pyramid pooling layers and pooling kernel size on apple leaf segmentation, we employ ResNet50 as the backbone network and consider both the number of pooling layers and kernel sizes as variable parameters. We design 11 distinct experimental settings, as outlined in **Table 4**. The first experimental scheme employs the original 4-scale pyramid pooling layers of PSPNet, consisting of [1 × 1,2 × 2,3 × 3,6 × 6]. Experiments 2-7 feature combinations of any two sizes from these four scales, while Experiments 8-11 incorporate combinations of any three sizes. By evaluating the segmentation performance across these configurations, we gain insights into the sensitivity of PSPNet to different pyramid pooling setups. This analysis aids in optimizing the network architecture specifically for the task of apple leaf segmentation. Our results suggest that both the presence and sizes of pooling layers substantially affect model performance on the apple leaf disease dataset. Optimal performance can be observed with pooling kernel sizes of [1 × 1,6 × 6].

**Table 4:** The pyramid pooling layer ablation experiment.

Experiment settings	The number of pools	Pool core size	mIoU (%)	mPA (%)	Precision (%)	Recall (%)
Setting 1	4	[1x1, 2x2, 3x3, 6x6]	94.57	97.10	97.59	97.10
Setting 2	2	[1x1,2x2]	92.04	95.44	95.13	95.44
Setting 3	2	[1x1,3x3]	93.39	96.49	96.36	96.49
Setting 4	2	[1x1,6x6]	**94.64**	**97.19**	**97.61**	**97.19**
Setting 5	2	[2x2,3x3]	93.58	96.41	97.01	96.41
Setting 6	2	[2x2,6x6]	93.42	96.60	96.98	96.60
Setting 7	2	[3x3,6x6]	93.31	96.43	96.82	96.43
Setting 8	3	[1x1,2x2,3x3]	93.60	96.77	96.83	96.77
Setting 9	3	[1x1,2x2,6x6]	93.54	96.60	97.32	96.60
Setting 10	3	[2x2,3x3,6x6]	92.19	95.53	96.12	95.53
Setting 11	3	[1x1,3x3,6x6]	93.98	96.85	97.34	96.85

Best values are in bold.

In **Table 5**, we conduct ablation studies on the deformable convolution layer. Specifically, we design 3 experiments that add the deformable convolution after the 1x1 pooling layer, after the 6x6 pooling layer, and after both 1x1 and 6x6 pooling layers, respectively. By comparing segmentation performance, we can validate the effectiveness of adding deformable convolutions to different levels of the feature pyramid, as well as investigate if concurrent deformation modeling on multiple levels can achieve complementary benefits. This ablation study provides insights on how to best incorporate deformable convolutions into the network architecture for enhanced modeling capability. Based on the data in **Tables 4**, **5**, we conclude that for the apple frog eye leaf spot dataset, excessive pyramid pooling layers are not advantageous. Best results were achieved with 1×1 and 6×6 kernel sizes and by incorporating deformable convolutions alongside the 6x6 pooling layer. This streamlined model structure eliminated redundancy and improved recognition performance, especially for occluded leaves. Compared to the original PSPNet, our modified model demonstrates improvements across all metrics, achieving scores of 97.74%, 98.82%, 98.90% and 98.82%, thereby confirming the benefits of integrating deformable convolutions.

**Table 5:** Ablation experiment of deformable convolution layer.

Experiment Setting	1x1	6x6	mIoU(%)	mPA(%)	Precision(%)	Recall(%)
Setting 1	✓		95.12	98.26	97.55	98.26
Setting 2		✓	**97.74**	**98.82**	**98.90**	**98.82**
Setting 3	✓	✓	94.09	96.37	97.55	96.37

Best values are in bold.

**Figure 8:**
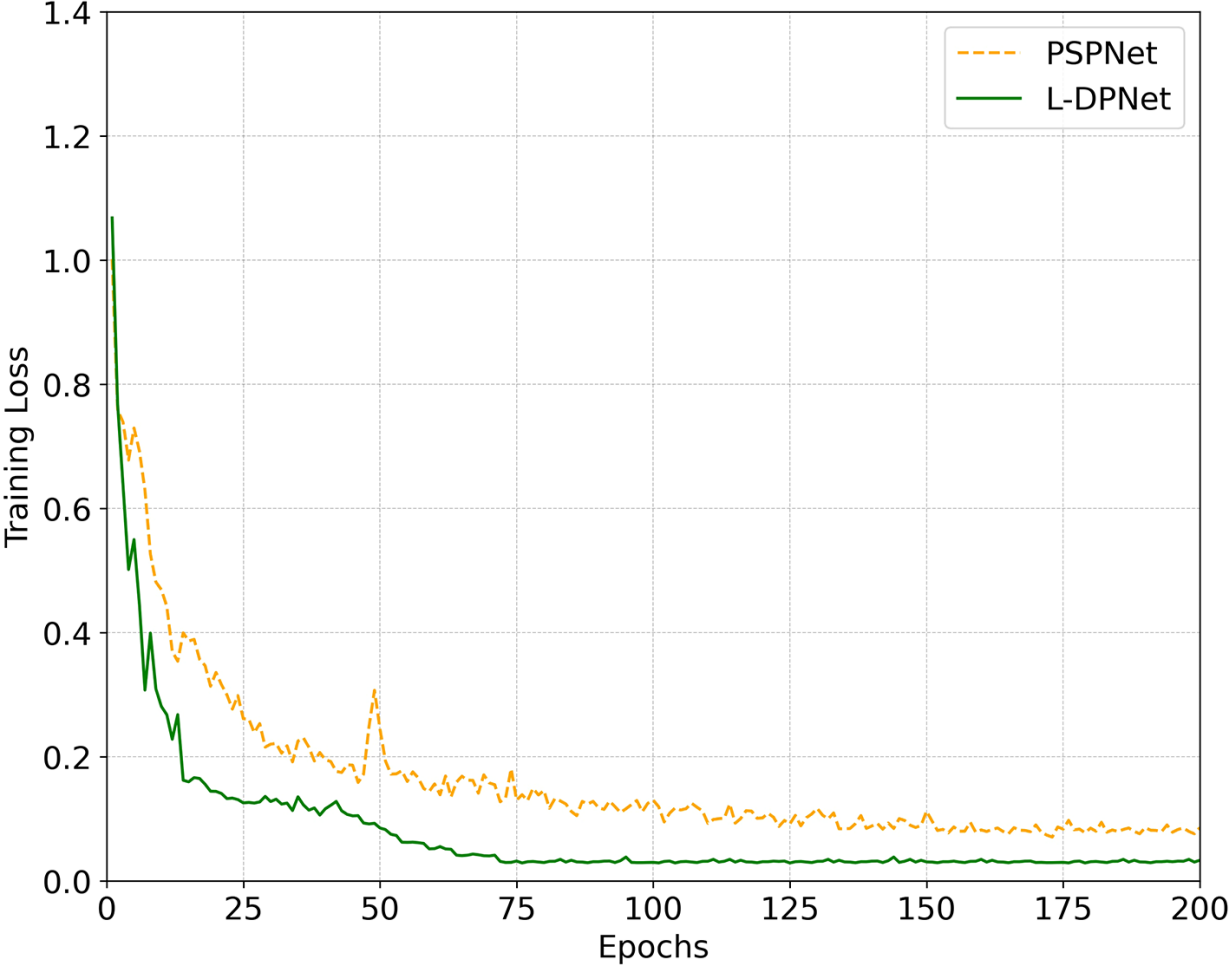
Convergence comparison between PSPNet and our proposed L-DPNet.

The change in training loss with iteration is depicted in **Figure 8**. This figure aims to compare the segmentation performance between the improved model, L-DPNet, and the original model, PSPNet. The graph reveals significant fluctuations in training loss during the early stages (0 to 75 iterations), followed by a gradual convergence. PSPNet shows higher loss and slower convergence, stabilizing after approximately 125 iterations. In contrast, L-DPNet demonstrates a more rapid decrease in loss during the 0 to 75 iteration range, with relative stability achieved between 75 to 200 iterations, indicating convergence.

In summary, the model’s performance has been optimized effectively through judicious adjustments to the pyramid pooling layers and the introduction of deformable convolutions. This has not only improved the accuracy of apple leaf recognition but has also significantly enhanced various evaluation metrics. These results strongly support the model’s utility for apple leaf disease segmentation tasks.

#### 3.3.2 Experimental analysis for disease segmentation

In the second stage of our work, we introduce a modified U-Net architecture, which we refer to as D-UNet, to handle the greater complexity in shape and size of disease spots compared to the apple leaves segmented in the first stage. In D-UNet, we incorporated batch normalization layers before each activation layer to mitigate overfitting. We also opted for bilinear interpolation over transposed convolutions for upsampling tasks in the decoder section. For D-UNet, we conduct four ablation studies: original UNet, replacing transpose convolution with bilinear interpolation only, using normalization layers only, and finally combining normalization with bilinear interpolation upsampling. Through comparing segmentation performance, we can validate the individual contribution of bilinear upsampling and normalization, as well as the combined effects when both enhancements are incorporated together. This systematic ablation study provides insights on the optimal configuration to improve upon the original UNet architecture.

The ablation study results for these D-UNet modifications are presented in **Table 6**. The results indicate that the introduction of batch normalization layers led to a 0.07% increase in mIoU, a 0.17% increase in mPA, a 1.34% increase in precision, and a 0.17% increase in recall. These enhancements are particularly beneficial for our task of segmenting apple frog eye leaf spots, where high pixel-level accuracy on unseen images is crucial.Furthermore, in D-UNet, the use of bilinear interpolation for upsampling in the decoder yielded more consistent and artifact-free results compared to transposed convolutions. This improvement was reflected in significant enhancements in all evaluation metrics: mIoU increased by 1.32%, mPA by 2.59%, precision by 1.35%, and recall by 2.59%.

**Table 6:** D-UNet model ablation experiment.

Experiment Setting	Normalized layer	Bilinear interpolation	mIoU(%)	mPA(%)	Precision(%)	Recall(%)
Experiment Setting 1			88.90	91.47	97.20	91.47
Experiment Setting 2		✓	89.86	92.37	98.20	92.37
Experiment Setting 3	✓		88.97	91.64	98.54	91.64
Experiment Setting 4	✓	✓	**90.29**	**94.23**	**99.89**	**94.23**

Best values are in bold.

#### 3.3.3 Experimental analysis for L-DPNet+D-UNet architecture

To validate the effectiveness of the proposed improvements in segmenting apple leaves and frog eye spots, we include comparisons with other state-of-the-art algorithms in our analysis to provide comprehensive evaluation (PUNet (Liu et al., 2022) and LD-DeepLabv3+ (Zhu et al., 2023)).

Both of these methods are specifically designed for disease severity estimation. PUNet employs PSPNet for leaf area extraction and U-Net for disease spot segmentation. LD-DeepLabv3+ uses an enhanced version of DeepLabv3+ to segment both the leaf and disease areas. Moreover, We included SOLOv2 (Wang et al., 2020b) and YOLACT (Bolya et al., 2019) to validate the effectiveness of one-stage instance segmentation methods in leaf and disease segmentation.SOLOv2 is an improved version of the original SOLO (Wang et al., 2020a) method. It is a one-stage instance segmentation approach that eliminates the need for anchor boxes and is known for its efficiency and accuracy. YOLACT is another one-stage instance segmentation method. It employs a mask coefficients to refine segmentation boundaries. To keep our comparison upto-date, we have included the latest version of the YOLO object detection algorithm, YOLOv8 (Jocher et al., 2023), which is known for its speed and accuracy. Although YOLO series methods are originally designed for object detection, we adapted it for our segmentation task. Specifically, we retained YOLOv8’s backbone, data augmentation, and training strategies, but replaced its detection head with YOLACT’s mask prediction head to better suit our segmentation needs.

As illustrated in **Table 7**, our approach surpasses competing methods across nearly all evaluation metrics, demonstrating its efficacy in segmenting both background and object classes, such as leaves and diseases. In general, two-stage algorithms like PUNet and LD-DeepLabv3+ achieve superior mIOU and mPA scores when compared to one-stage counterparts like SOLOv2 and YOLACT. Although YOLOv8 excels over SOLOv2 and YOLACT in several aspects, it doesn’t quite match the performance of two-stage models. This enhanced precision in two-stage methods likely arises from their step-by-step procedure: initially identifying the leaf area and subsequently focusing on disease spot segmentation.

**Table 7:** Performance of apple leaf and frog eye spot segmentation under different model architectures.

Methods	class	IoU(%)	PA(%)	Precision(%)	Recall(%)	mIoU(%)	mPA(%)	mPrecision(%)	mRecall(%)
PUNet	Background Leaf Disease	97.89 92.45	98.96 94.78	99.02 95.02	98.96 94.78	89.44	92.43	92.65	92.43
		77.98	83.56	83.90	83.56				
LD-DeepLabv3+	Background Leaf Disease	97.55 91.58	97.89 94.01	98.78 94.82	97.89 94.01	88.94	91.70	92.46	91.66
		77.69	83.20	83.77	83.09				
SOLOv2	Background Leaf Disease	97.12 91.05	97.35 93.47	97.78 93.30	97.52 93.47	88.35	91.08	91.33	91.14
		76.89	82.43	82.90	82.43				
YOLACT	Background Leaf Disease	96.93 90.10	97.06 92.90	97.28 92.92	97.06 92.90	87.87	90.71	90.98	90.71
		76.59	82.18	82.75	82.18				
YOLOv8	Background Leaf Disease	97.19 91.26	97.52 93.58	97.98 93.49	97.35 93.58	88.60	91.40	91.71	91.38
		77.34	83.09	83.67	83.20				
Ours	Background Leaf Disease	**98.81** **96.67**	**98.98** **98.66**	**99.50** **98.30**	**98.98** **98.66**	**91.27**	**94.32**	**94.19**	**94.32**
		**78.33**	**85.32**	**84.77**	**85.32**				

Best values are in bold.

#### 3.3.4 Experimental analysis for the estimation of disease severity levels

The severity predicted by the trained model was compared with manually labeled severity levels for 138 images in the test set to calculate the model’s classification accuracy. The results are presented in **Table 8**, which lists the number of datasets used for testing and validating severity levels, the number of correctly classified images, and the accuracy ratio derived from these two values.

**Table 8:** Performance for disease severity classification.

Disease Classification	Number of dataset	Correct Grading	Precision(%)
Level 1	38	37	97.37
Level 2	30	29	96.67
Level 3	30	29	96.67
Level 4	30	28	93.33
Level 5	10	9	90.00
Total	138	132	94.81

**Figure 9:**
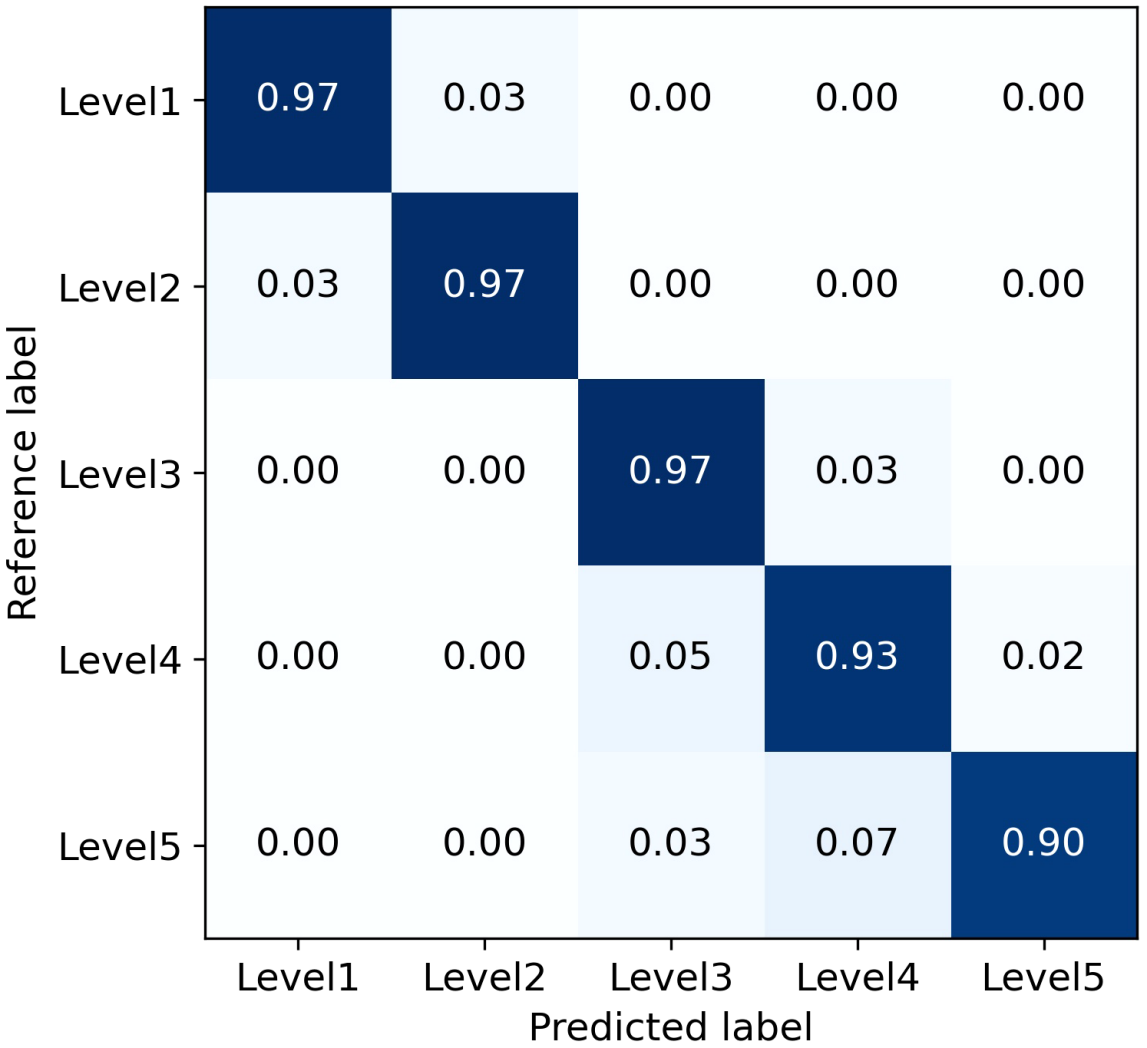
Confusion matrix for classification of apple frog eye leaf spot severity.

The average Precision for all levels and for levels 1-3 are 94.81% and 96.90%, respectively. Although the combined L-DPNet+D-UNet architecture achieved high classification accuracy in estimating the severity of apple frog-eye leaf spot disease, we can observe a tendency for Level 1 samples to be misclassified as Level 2, as shown in **Figure 9**. A likely reason for this is the similarity in the areas of lesions for Levels 1 and 2, which can lead to misdiagnosis. Generally, misclassified samples are confused with labels that are adjacent in severity, which is mainly due to segmentation errors but remains within an acceptable margin of error.

However, the accuracy for classifying severity Levels 4 and 5 is lower than that for Levels 1-3. This discrepancy is attributed to the higher proportion of Levels 1-3 in the dataset used for training, thereby limiting the model’s proficiency in recognizing Levels 4 and 5. The performance of the proposed model could be enhanced by incorporating datasets that cover a broader range of apple frog-eye leaf spot disease severity levels. Additionally, leveraging prior knowledge from fields like plant protection, along with advanced computer vision techniques, could contribute to a more effective severity assessment process.

#### 3.3.5 Visual Evaluation of L-DPNet and D-UNet models

**Figure 10:**
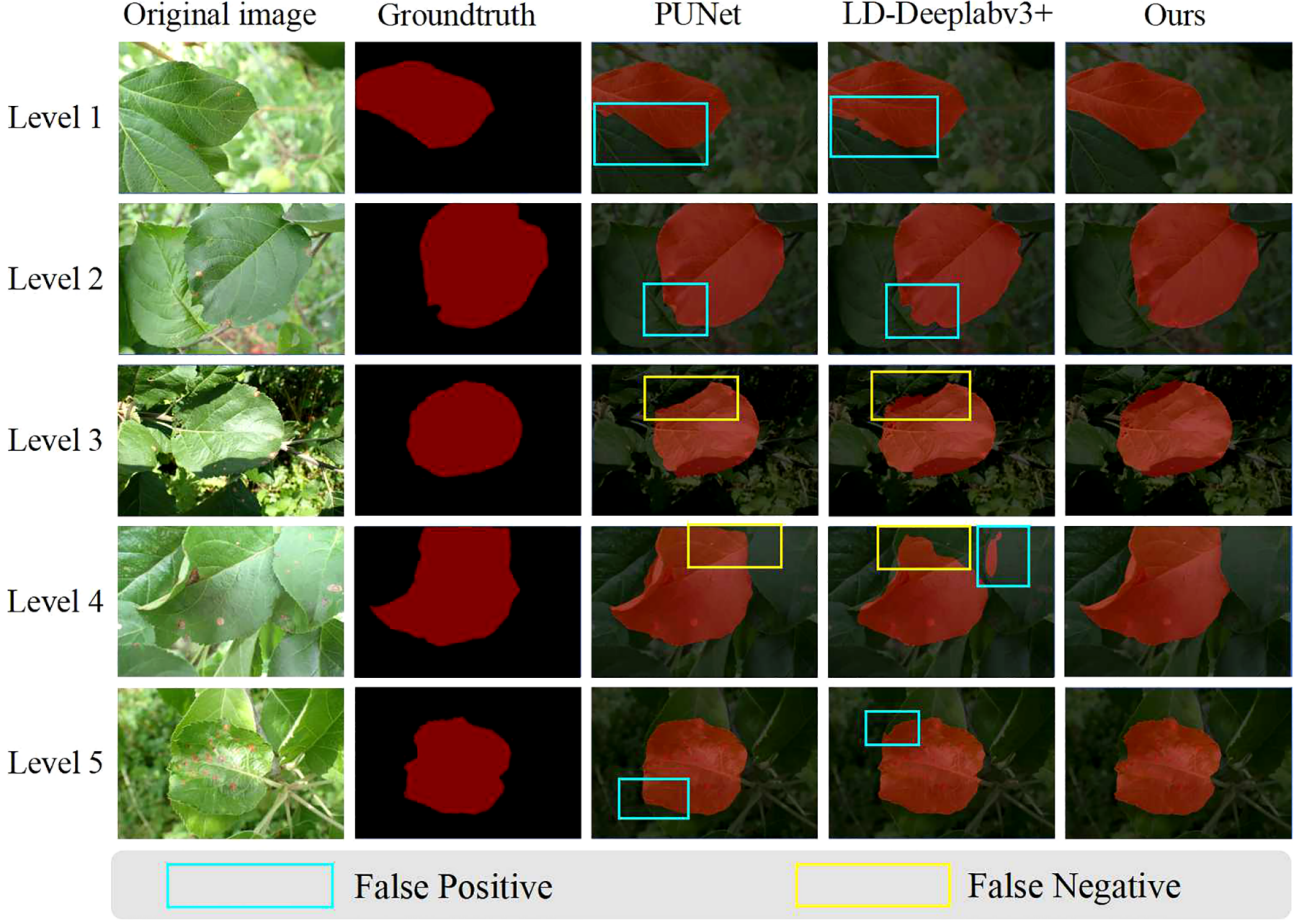
Visual results of leaf segmentation using two-stage methods. This figure compares the predictions made by PUNet, LD-DeepLabV3+, and our approach, L-DPNet+D-UNet, for apple leaves. Areas marked with blue boxes indicate false positives, while areas marked with yellow boxes indicate false negatives.

**Figure 11:**
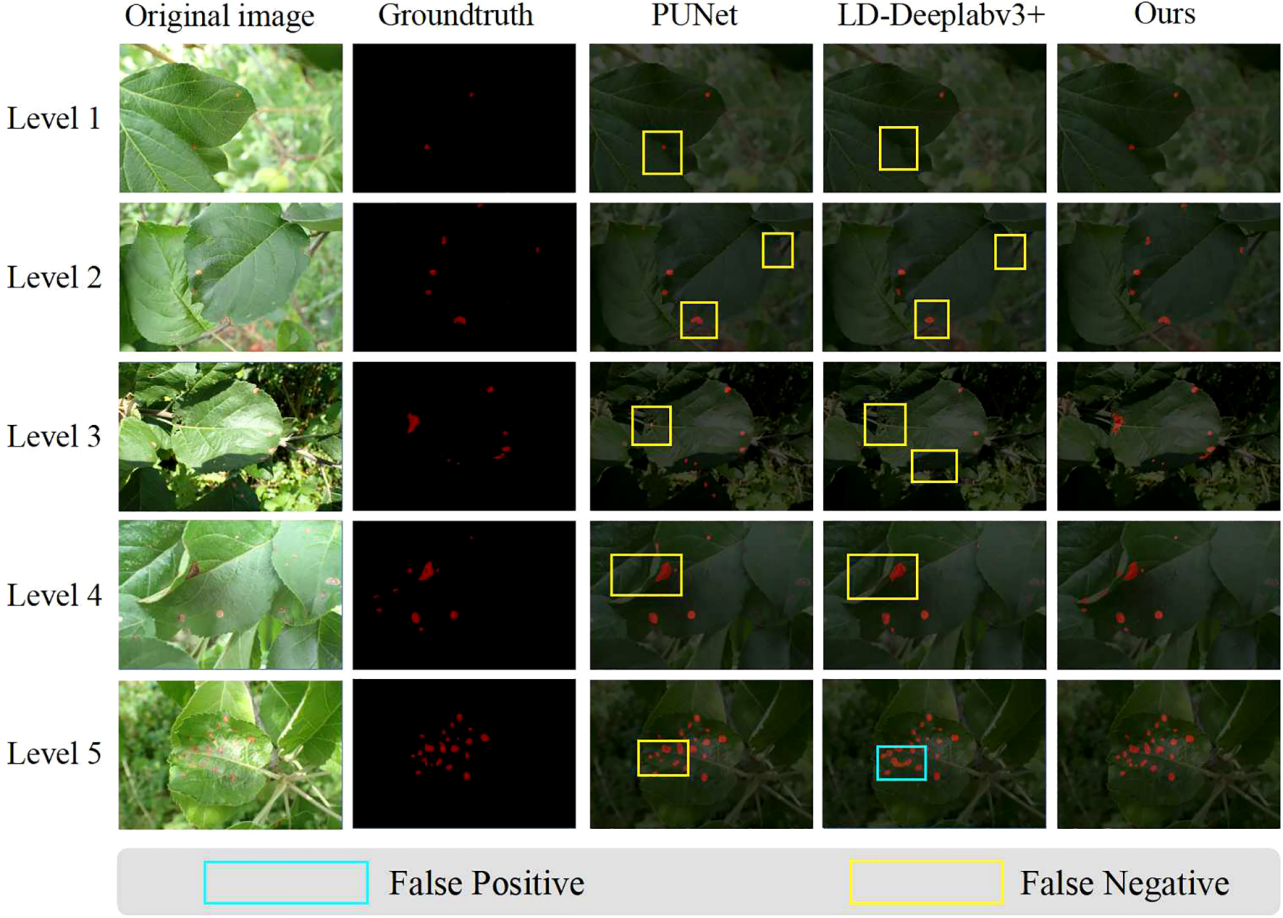
Visual results of disease spot segmentation using two-stage methods. This figure compares the disease spot predictions made by PUNet, LD-DeepLabV3+, and our approach, L-DPNet+D-UNet. Areas marked with blue boxes indicate false positives, while areas marked with yellow boxes indicate false negatives.

**Figure 12:**
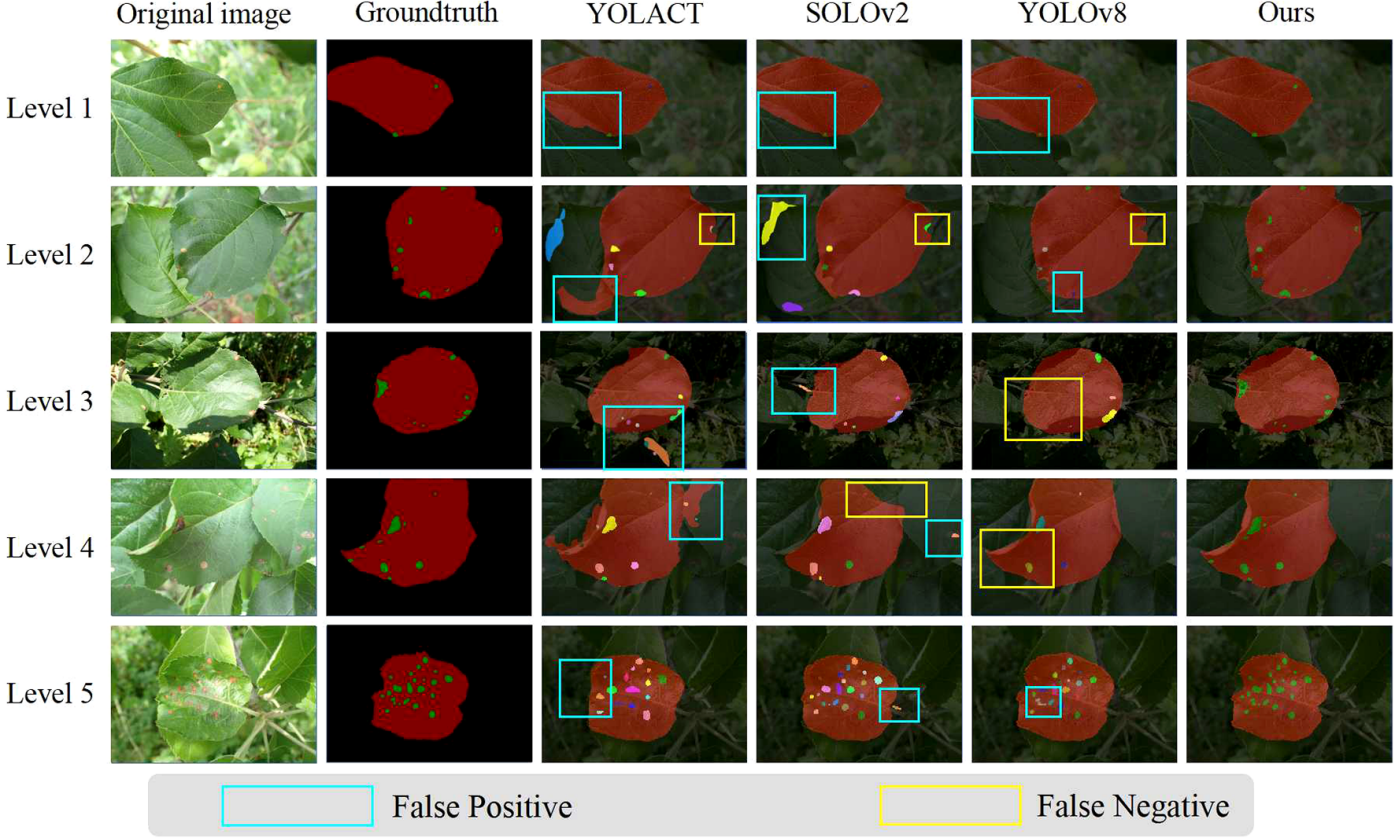
Visual comparison between our two-stage method and one-stage methods, using different color masks for different instances. This figure compares the predictions made by YOLACT,SOLOv2,YOLOv8 and our approach, L-DPNet+D-UNet, for apple leaves and disease spots. Areas marked with blue boxes indicate false positives, while areas marked with yellow boxes indicate false negatives.

**Visualization of segmentation results:** We have expanded our analysis to include a more nuanced evaluation of the segmentation results, focusing on both leaves and lesions. Considering that we have conducted comparisons with representative two-stage and one-stage methods, **Figures 10**, **11** respectively display the visual results of leaf and disease spot segmentation using two-stage methods. Meanwhile, **Figure 12** presents the comparative results with one-stage segmentation methods. Specifically, we discuss the performance of different methods under five distinct scenarios:

As shown in Level 1, 4 and 5 of **Figure 10**, when leaf overlap exists, both PUNet and LD-DeepLabv3+ exhibit recognition errors to some extent. In Level 1 and Level 5, the leaf edges are over-segmented, while in Level 4, the overlapping leaf edges are under-segmented. In contrast, our proposed model can accurately segment the leaf edges, laying the foundation for subsequent lesion segmentation. In Level 3 of **Figure 10**, the shadow areas formed by illumination lead to under-segmentation of leaves in PUNet and LD-DeepLabv3+ which fail to identify the shadowed regions. Comparatively, our improved model can better restore the complete leaf shapes when dealing with illumination variations. When natural edge defects (**Figure 10** Level 2) caused by leaf curling exist, PUNet wrongly recognizes the missing edges as complete leaf regions. As for edge defects resulting from lesions (**Figure 10** Level 2), LD-DeepLabv3+ cannot effectively identify such edges. Our model can effectively distinguish between these two edge cases and produce superior segmentation. On leaves with mild diseases, tiny lesion spots often appear (**Figure 11** Level 1). PUNet can identify small spots but fails to fully segment them, which will affect the final severity assessment. LD-DeepLabv3+, on the other hand, directly misses some lesions (**Figure 11** Level 4). In contrast, our D-UNet can not only accurately locate the spots, but also segment them precisely. When dense spots occur (**Figure 12** Level 4), PUNet will erroneously group adjacent spots into a single large one, and also fails to segment small spots. LD-DeepLabv3+ causes spot merging. Our model achieves finer segmentation of dense disease spots, which further improves the accuracy of severity estimation.

Additionally, owing to the capability of two-stage methods to accurately localize objects with varying scales, such as leaves and disease spots, our proposed two-stage method results in fewer false positives for both leaf (**Figure 12** Level 1) and disease spot areas (**Figure 12** Level 5), as well as fewer false negatives for leaf (**Figure 12** Level 2) and disease spot regions (**Figure 12** Level 4).

**Visualization of feature maps:** To elucidate the differences in leaf segmentation capabilities between the original PSPNet and the improved L-DPNet, we visualized the feature maps of both models. The results are displayed in **Supplementary Figure 3**. In the feature map of the original PSPNet, as seen in **Supplementary Figure 3A**, the extracted features appear relatively blurry, providing only a rough localization of the leaves and limited detail. In contrast, the feature map of L-DPNet, shown in **Supplementary Figure 3B**, demonstrates significant improvements. By incorporating deformable convolution kernels that adaptively adjust their shape and size, L-DPNet is better attuned to the leaves’ shape and structure. This results in feature maps with clearer leaf edges and enhanced detail, effectively differentiating the apple leaves from the background.

## 4 Conclusion

In this study, we introduced a two-stage approach using L-DPNet and D-UNet for automated apple disease severity assessment. The first stage employs L-DPNet, achieving a leaf segmentation accuracy of 98.30%. This model is particularly effective in separating apple leaves from complex natural backgrounds, setting the foundation for subsequent disease spot segmentation. The second stage utilizes D-UNet, which builds upon the VGG16 architecture and includes batch normalization and bilinear interpolation to achieve a lesion segmentation accuracy of 84.77%. Finally, our models contribute to an overall severity classification accuracy of 94.81% across five severity levels. Compared to individual models, our collaborative framework demonstrates stronger adaptability to complex backgrounds and accurate identification of fine details. Segmentation-based severity computation enables more delicate and continuous disease quantification, guiding precision treatment. The proposed framework has the potential to be integrated into orchard inspection robots or intelligent monitoring systems for early disease detection and treatment. Our upcoming research will focus on optimizing the computational efficiency of the model without compromising its accuracy. We also aim to extend the model’s capabilities to include dynamic monitoring of leaf areas and the recognition of multiple diseases on the same leaf.

## Data availability statement

The original contributions presented in the study are included in the article/**Supplementary Material**. Further inquiries can be directed to the corresponding author.

## Author contributions

HC: Conceptualization, Methodology, Writing – original draft. HF: Data curation, Formal analysis, Software, Writing – original draft. YZ: Formal analysis, Validation, Writing – review & editing, Data curation. JC: Formal analysis, Validation, Writing – review & editing. BL: Funding acquisition, Methodology, Project administration, Writing – review & editing.

## Footnotes

1
https://www.kaggle.com/competitions/plant-pathology-2020-fgvc7/data

2
https://www.kaggle.com/competitions/plant-pathology-2021-fgvc8/data
