# Supplementary material for: Deep learning architectures for diagnosing the severity of apple frog-eye leaf spot disease in complex backgrounds
Source: Front Plant Sci. 2024 Jan 8;14:1289497. doi: 10.3389/fpls.2023.1289497 (PMC10800469; doi:10.3389/fpls.2023.1289497)
Supplement: Supplementary file 1 [file DataSheet_1.pdf]

Supplementary Material

1 SUPPLEMENTARY TABLES AND FIGURES

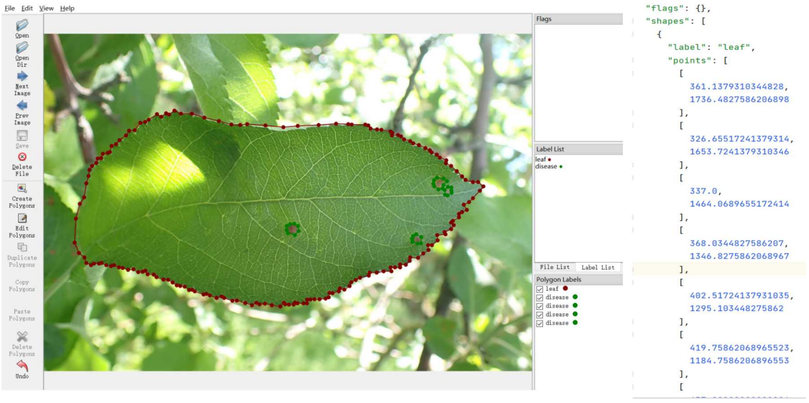

Figure S1. Labelme image annotation and file content.

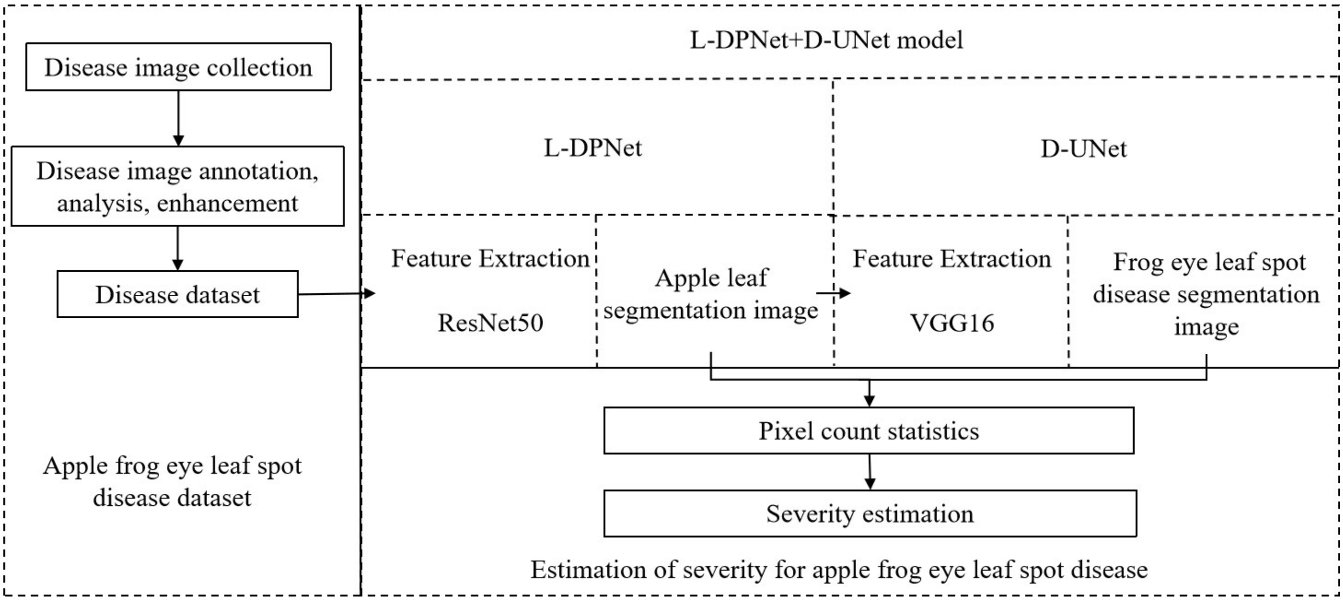

Figure S2. Flow chart of L-DPNet+D-UNet model of the proposed method.

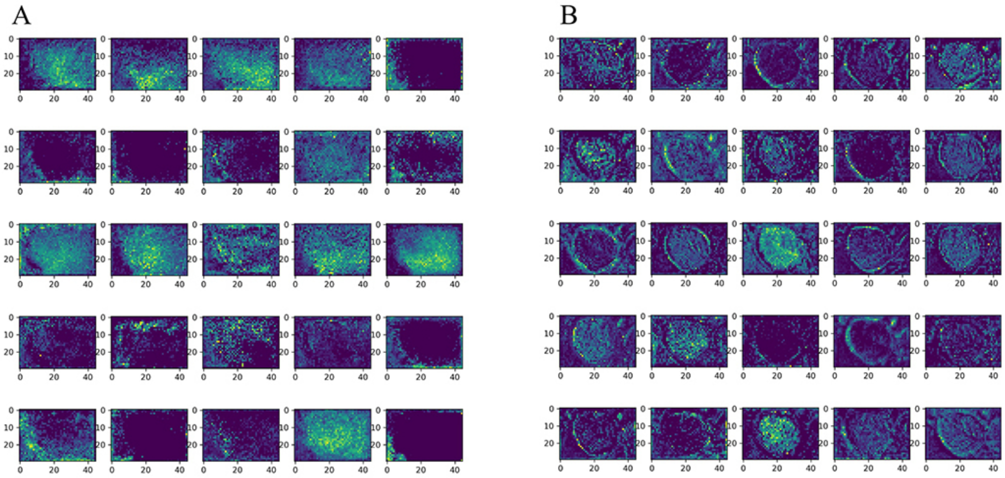

Figure S3. Visualization of feature layers: **(A)** Standard convolution network; **(B)** Deformable convolution network.

Table S1. Structure of the D-UNet network model.

Module	Contracting	Kernel size&stride	Output size	Module	Decoding	Kernel size &stride	Output size
Encoder 1	Conv1	3x3,1	64x512x512	En-Decoder	Conv11	3x3,1	512x32x32
	BN	-	64x512x512		BN	-	512x32x32
	Relu	-	64x512x512		Relu	-	512x32x32
	Conv2	3x3,1	64x512x512		Conv12	3x3,1	512x32x32
	BN	-	64x512x512		BN	-	512x32x32
	Relu	-	64x512x512		Relu	-	512x32x32
	Maxpooling	2x2,2	64x256x256		Conv13	3x3,1	512x32x32
Encoder 2	Conv3	3x3,1	128x256x256		BN	-	512x32x32
	BN	-	128x256x256		Relu	-	512x32x32
	Relu	-	128x256x256	Decoder 1	UpsamplingBilinear2d	scale_factor=2	512x64x64
	Conv4	3x3,1	128x256x256		Concat	-	1024x64x64
	BN	-	128x256x256		Conv14	3x3,1	512x64x64
	Relu	-	128x256x256		Conv15	3x3,1	512x64x64
	Maxpooling	2x2,2	128x128x128		Relu	-	512x64x64
Encoder 3	Conv5	3x3,1	256x256x128	Decoder 2	UpsamplingBilinear2d	scale_factor=2	256x128x128
	BN	-	256x256x128		Concat	-	768x128x128
	Relu	-	256x256x128		Conv16	3x3,1	256x128x128
	Conv6	3x3,1	256x256x128		Conv17	3x3,1	256x128x128
	BN	-	256x256x128		Relu	-	256x128x128
	Relu	-	256x256x128	Decoder 3	UpsamplingBilinear2d	scale_factor=2	128x256x256
	Conv7	3x3,1	256x256x128		Concat	-	384x256x256
	BN	-	256x256x128		Conv18	3x3,1	128x256x256
	Relu	-	256x256x128		Conv19	3x3,1	128x256x256
	Maxpooling	2x2,2	256x64x64		Relu	-	128x256x256
Encoder 4	Conv8	3x3,1	512x512x64	Decoder 4	UpsamplingBilinear2d	scale_factor=2	64x512x512
	BN	-	512x512x64		Concat	-	192x512x512
	Relu	-	512x512x64		Conv20	3x3,1	64x512x512
	Conv9	3x3,1	512x512x64		Conv21	3x3,1	64x512x512
	BN	-	512x512x64		Relu	-	64x512x512
	Relu	-	512x512x64	Classifying	Conv22	1x1,1	3x512x512
	Conv10	3x3,1	512x512x64				
	BN	-	512x512x64				
	Relu	-	512x512x64				
	Maxpooling	2x2,2	512x32x32				
